# Surgery for primary tumor benefits survival for breast cancer patients with bone metastases: a large cohort retrospective study

**DOI:** 10.1186/s12885-021-07964-9

**Published:** 2021-03-04

**Authors:** Zhangheng Huang, Xin Zhou, Yuexin Tong, Lujian Zhu, Ruhan Zhao, Xiaohui Huang

**Affiliations:** 1 Department of Clinical Medicine, Hangzhou Medical College, 481 Binwen Road, Hangzhou, Zhejiang Province China; 2 Chengde Medical University, Chengde, Hebei Province China; 3 The First Affiliated Hospital of Wenzhou Medical University, Wenzhou, Zhejiang Province China

**Keywords:** Surgery, Breast cancer, Bone metastases, Nomogram, SEER, Prognosis

## Abstract

**Background:**

The role of surgery for the primary tumor in breast cancer patients with bone metastases (BM) remains unclear. The purpose of this study was to determine the impact of surgery for the primary tumor in breast cancer patients with BM and to develop prognostic nomograms to predict the overall survival (OS) of breast cancer patients with BM.

**Methods:**

A total of 3956 breast cancer patients with BM from the Surveillance, Epidemiology, and End Results database between 2010 and 2016 were included. Propensity score matching (PSM) was used to eliminate the bias between the surgery and non-surgery groups. The Kaplan-Meier analysis and the log-rank test were performed to compare the OS between two groups. Cox proportional risk regression models were used to identify independent prognostic factors. Two nomograms were constructed for predicting the OS of patients in the surgery and non-surgery groups, respectively. In addition, calibration curve, receiver operating characteristic (ROC) curve, and decision curve analysis (DCA) were used to evaluate the performance of nomograms.

**Result:**

The survival analysis showed that the surgery of the primary tumor significantly improved the OS for breast cancer patients with BM. Based on independent prognostic factors, separate nomograms were constructed for the surgery and non-surgery groups. The calibration and ROC curves of these nomograms indicated that both two models have high predictive accuracy, with the area under the curve values ≥0.700 on both the training and validation cohorts. Moreover, DCA showed that nomograms have strong clinical utility. Based on the results of the X-tile analysis, all patients were classified in the low-risk-of-death subgroup had a better prognosis.

**Conclusion:**

The surgery of the primary tumor may provide survival benefits for breast cancer patients with BM. Furthermore, these prognostic nomograms we constructed may be used as a tool to accurately assess the long-term prognosis of patients and help clinicians to develop individualized treatment strategies.

## Introduction

Despite rapid advances in endocrine and targeted therapy in recent years, breast cancer is still one of the leading causes of cancer death in women [1]. Bone is the most common site of metastasis in breast cancer patients, and up to 6% of all breast cancer patients already have bone metastases (BM) at the time of initial diagnosis [2, 3]. BM often results in skeletal-related events, including spinal cord compression, pathological fractures, hypercalcemia, and severe pain [4]. These complications negatively affect patients’ mobility and mental status, with significant reductions in their quality of life [5, 6].

It is well known that surgery is a common means of treating early-stage breast cancer. Currently, palliative treatments, such as chemotherapy, endocrine therapy, and targeted therapy, are used to improve survival, control tumor burden, reduce cancer-related symptoms and maintain quality of life for breast cancer patients with BM [7]. Nevertheless, the effectiveness of palliative care in terms of survival is poor, as even high-dose chemotherapy and stem cell transplantation do not improve the survival of these patients [8]. Traditionally, surgery has not been recommended for patients with distant metastases. Several retrospective studies have shown that patients with metastatic breast cancer, those who undergo surgery for the primary tumor survive longer than those who do not undergo surgery for the primary tumor [9–11]. Conversely, it has also been reported that for stage IV breast cancer, surgery of the primary tumor may lead to enhanced metastatic spread and adversely affect prognosis by suppressing anti-metastatic cell-mediated immunity and increasing pro-angiogenic factor production [12–14].

To better study the potential benefits and beneficiary populations of the surgery, we will focus on a more specific and limited set of diseases. Also, the difference in the location of distant metastases from breast cancer can make a difference in overall survival (OS). The median OS of breast cancer patients with liver metastasis is 6 months, the median OS of breast cancer patients with lung metastasis is 14.1 months, while the median OS of breast cancer patients with BM is 28 months [15–17]. Due to the fact that BM is most common in breast cancer and tend to have longer survival than other single metastases, these patients are more likely to undergo surgery for the primary tumor [18]. Therefore, it is of great clinical importance to study the role of surgery of the primary tumor in breast cancer patients with BM.

The study used data from the Surveillance, Epidemiology, and End Results (SEER) database to investigate the value of primary tumor surgery in breast cancer patients with BM, and to identify independent prognostic factors associated with survival in patients who underwent surgery and those who did not, respectively. Furthermore, on this basis, we constructed nomograms for predicting the prognosis of breast cancer patients with BM who underwent surgery or did not undergo surgery.

## Methods

### Patient selection

This population-based retrospective study used data from the SEER database. The SEER program consists of 18 population-based cancer registries that collect statistical, oncological, diagnostic, and treatment information on approximately 28% of the United States population. The data included in this study were downloaded from the SEER*Stat software (version 8.3.6). As the SEER database did not record distant metastases before 2010, our study only considered breast cancer patients with BM between 2010 and 2016. The inclusion criteria were as follows: (1) primary breast cancer patients, (2) patients with BM, (3) patients with complete clinicopathologic features, demographic data, and follow information. Besides, patients confirmed by autopsy or death were excluded. Ultimately, we selected 3956 breast cancer patients with BM from 447,929 breast cancer patients to form the study cohort.

### Variable definitions

Patients’ demographic characteristics (age, sex, race, insurance status, and marital status), disease characteristics (primary site, laterality, histological type, grade, T stage, N stage, tumor size, breast subtype, and distant metastatic sites), treatment modalities (radiotherapy, chemotherapy, and surgery), survival time, and vital status were incorporated in our study. The optimal cutoff values for age in terms of OS were determined by X-tile software (Yale University, New Haven, CT, USA), and patients were divided into 3 groups (< 51, 51–78, and > 78 years). In terms of tumor size, we divided the patients into 3 groups (< 2, 2–5, and > 5 cm). The primary site is defined according to the International Classification of Diseases for Oncology codes: central portion of breast (C50.1), upper-inner quadrant of the breast(C50.2), lower-inner quadrant of the breast (C50.3), upper-outer quadrant of the breast (C50.4),lower-outer quadrant of the breast (C50.5), and others (C50.0, C50.6, C50.8, and C50.9). The patient’s histological type was classified as ductal carcinoma, lobular carcinoma, and others. The degree of tumor differentiation was divided into four groups: grade I, grade II, grade III, and grade IV. Patients were divided into two groups, surgery (breast-conserving surgery (BCS) and mastectomy) and non-surgery, depending on the specific surgical treatment. All cases in this study were staged using the 7th edition TNM staging system.

### Statistical analysis

The characteristics of the surgery and non-surgery groups were compared using the Chi-squared test. To eliminate bias between the surgery and non-surgery groups, we performed the propensity score matching (PSM) analysis. In the survival analysis, the primary endpoint of our study was OS, which was defined as the interval between the day of diagnosis and the day of death due to any causes or the date of the last follow-up. Kaplan-Meier analysis and the log-rank test were used to compare the OS of patients in the surgery and non-surgery groups after PSM. In addition, we compared the OS of patients who underwent BCS with those who underwent mastectomy after PSM.

Patients in the surgery and non-surgery groups before PSM were randomized in a 7:3 ratio into a training cohort and a validation cohort, respectively, and the classification process was performed in the R software. Univariate and multivariate Cox proportional hazards regression analyses of two groups of the training cohort were used to identify independent prognostic factors. The nomograms used to predict the OS of patients in the surgery and non-surgery groups were constructed separately based on corresponding independent prognostic factors. The discrimination of the nomograms was evaluated using receiver operating characteristic (ROC) curves and the area under the curve (AUC). Moreover, we used calibration curves to measure the agreement between predicted and actual outcomes. The clinical application value of the nomograms was evaluated by decision curve analysis (DCA). The optimal cutoff value for the scores from nomograms in terms of OS was determined by X-tile software, and patients were divided into three groups (low risk, mid risk, and high risk). To further validate the accuracy and performance of the nomogram model, we also evaluated these nomograms in the validation cohort. This study used SPSS 25.0 (NY, USA) and R software (version 3.6.1) for statistical analysis. In the present study, a *P* value< 0.05(two sides) was identified as statistical significance.

## Results

### Baseline characteristics of breast cancer patients with BM before and after PSM

**Fig. 1:**
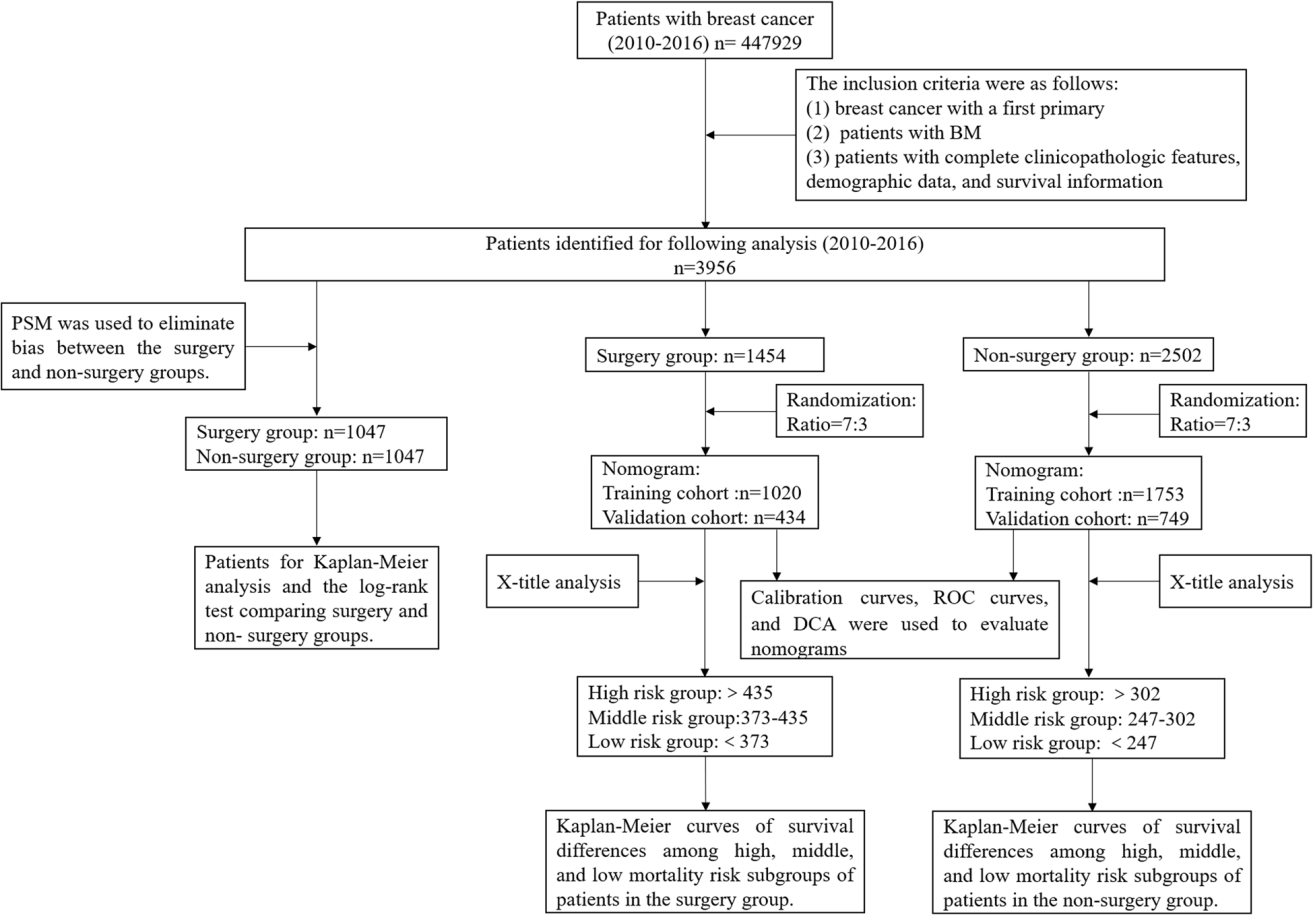
Flow diagram of patient selection and study development. BM: bone metastases, PSM: propensity score matching, ROC: receiver operating characteristic, DCA: decision curve analysis

The workflow of our study is illustrated in the Fig. 1. From 2010 to 2016, 447,929 breast cancer patients were identified in the SEER database, 3956 of whom met our research criteria. A total of 1454 patients underwent surgery and another 2502 did not. As shown in Table 1, there were significant differences in most of the baseline characteristics between patients in the surgery and non-surgery groups, such as age, histological type, grade, T stage, N stage, radiotherapy, chemotherapy, brain metastasis, liver metastasis, lung metastasis, tumor size, breast subtype, insurance status, and marital status. A total of 2094 patients were matched between the surgery and non-surgery groups after PSM, while all variables were balanced between these two groups (Table 1).

**Table 1:** Baseline characteristics of all BC patients with BM and PSM patients

Category	All patients	Non-surgery (%)	P value	PSM patients	Non-surgery (%)	P value
	Surgery (%)			Surgery (%)		
**Age**			0.000			0.467
< 51	422 (29.0)	610 (24.4)		281 (28.6)	282 (26.9)	
51–78	916 (63.0)	1599 (63.9)		676 (64.6)	659 (62.9)	
> 78	116 (8.0)	293 (11.7)		90 (8.6)	106 (10.1)	
**Race**			0.881			0.153
Black	218 (15.0)	390 (15.6)		160 (15.3)	142 (13.6)	
Other	114 (7.8)	196 (7.8)		72 (6.9)	93 (8.9)	
White	1122 (77.2)	1916 (76.6)		815 (77.8)	812 (77.6)	
**Sex**			0.067			0.394
Female	1426 (98.1)	2472 (98.8)		1032 (98.6)	1027 (98.1)	
Male	28 (1.9)	30 (1.2)		15 (1.4)	20 (1.9)	
**Primary site**			0.534			0.988
Central portion	157 (10.8)	241 (9.6)		107 (10.2)	104 (9.9)	
Upper-inner	149 (10.2)	233 (9.3)		106 (10.1)	103 (9.8)	
Lower-inner	75 (5.2)	145 (5.8)		58 (5.5)	62 (5.9)	
Upper-outer	495 (34.0)	906 (36.2)		366 (35.0)	377 (36.0)	
Lower-outer	114 (7.8)	196 (7.8)		79 (7.5)	81 (7.7)	
Others	464 (31.9)	781 (31.2)		331 (31.6)	320 (30.6)	
**Laterality**			0.512			0.457
Left	772 (53.1)	1293 (51.6)		548 (52.3)	565 (54.0)	
Right	682 (46.9)	1209 (48.3)		499 (47.7)	482 (46.0)	
**Histological type**			0.007			0.931
Ductal	1143 (78.6)	1971 (78.8)		841 (80.3)	845 (80.7)	
Lobular	264 (18.2)	402 (16.1)		170 (16.2)	169 (16.1)	
Others	47 (3.2)	129 (5.2)		36 (3.4)	33 (3.2)	
**Grade**			0.000			0.550
I	111 (7.6)	255 (10.2)		87 (8.3)	90 (8.6)	
II	593 (40.8)	1261 (50.4)		478 (45.7)	455 (43.5)	
III	747 (51.4)	975 (39.0)		479 (45.7)	501 (47.9)	
IV	3 (0.2)	11 (0.4)		3 (0.3)	1 (0.01)	
**T stage**			0.000			0.567
T1	178 (12.2)	371 (14.8)		133 (12.7)	134 (12.8)	
T2	650 (44.7)	971 (38.8)		458 (43.7)	475 (45.4)	
T3	307 (21.1)	471 (18.8)		206 (19.7)	181 (17.3)	
T4	319 (21.9)	689 (27.5)		250 (23.9)	257 (25.4)	
**N stage**			0.000			0.544
N0 + N1	779 (53.0)	2069 (82.7)		715 (68.3)	702 (67.0)	
N2 + N3	684 (47.0)	433 (17.3)		332 (31.7)	345 (33.0)	
**Radiotherapy**			0.000			0.861
No	686 (47.2)	1605 (64.1)		556 (53.1)	560 (53.5)	
Yes	768 (52.8)	897 (35.9)		491 (46.9)	487 (46.5)	
**Chemotherapy**			0.000			0.249
No	519 (35.7)	1235 (49.4)		422 (40.3)	448 (42.8)	
Yes	935 (64.3)	1267 (50.6)		625 (59.7)	599 (57.2)	
**Brain metastasis**			0.000			0.404
No	1419 (97.6)	2322 (92.8)		1014 (96.8)	1007 (96.2)	
Yes	35 (2.4)	180 (7.2)		33 (3.2)	40 (3.8)	
**Liver metastasis**			0.000			0.448
No	1261 (86.7)	1858 (74.3)		877 (83.8)	864 (82.5)	
Yes	193 (13.3)	644 (25.7)		170 (16.2)	183 (17.5)	
**Lung metastasis**			0.000			0.162
No	1240 (85.3)	1817 (72.6)		863 (82.4)	838 (80.0)	
Yes	214 (14.7)	685 (27.4)		184 (17.6)	209 (20.0)	
**Tumor size, cm**			0.004			0.829
< 2	157 (10.8)	362 (14.5)		124 (11.8)	125 (11.9)	
2–5	796 (54.7)	1333 (53.3)		572 (54.6)	584 (55.8)	
> 5	501 (34.5)	807 (32.3)		351 (33.5)	338 (32.3)	
**Breast subtype**			0.023			0.889
HR−/HER2+	91 (6.3)	153 (6.1)		62 (5.9)	69 (6.6)	
HR+/HER2-	963 (66.2)	1703 (68.1)		705 (67.3)	695 (66.4)	
HR+/HER2+	231 (15.9)	429 (17.1)		171 (16.3)	168 (16.0)	
HR−/HER2-	169 (11.6)	217 (8.7)		109 (10.4)	115 (11.0)	
**Insurance status**			0.000			0.128
Uninsured	32 (2.2)	123 (4.9)		27 (2.5)	17 (1.6)	
Insured	1422 (97.8)	2379 (95.1)		1020 (97.4)	1030 (98.4)	
**Marital status**			0.000			0.760
Unmarried	688 (47.3)	1354 (54.1)		529 (50.5)	522 (49.9)	
Married	766 (52.7)	1148 (45.9)		518 (49.5)	525 (50.1)	

*BC* breast cancer, *BM* bone metastases, *PSM* propensity score matching

### Survival benefit analysis of patients in the surgery and non-surgery groups after PSM

**Fig. 2:**
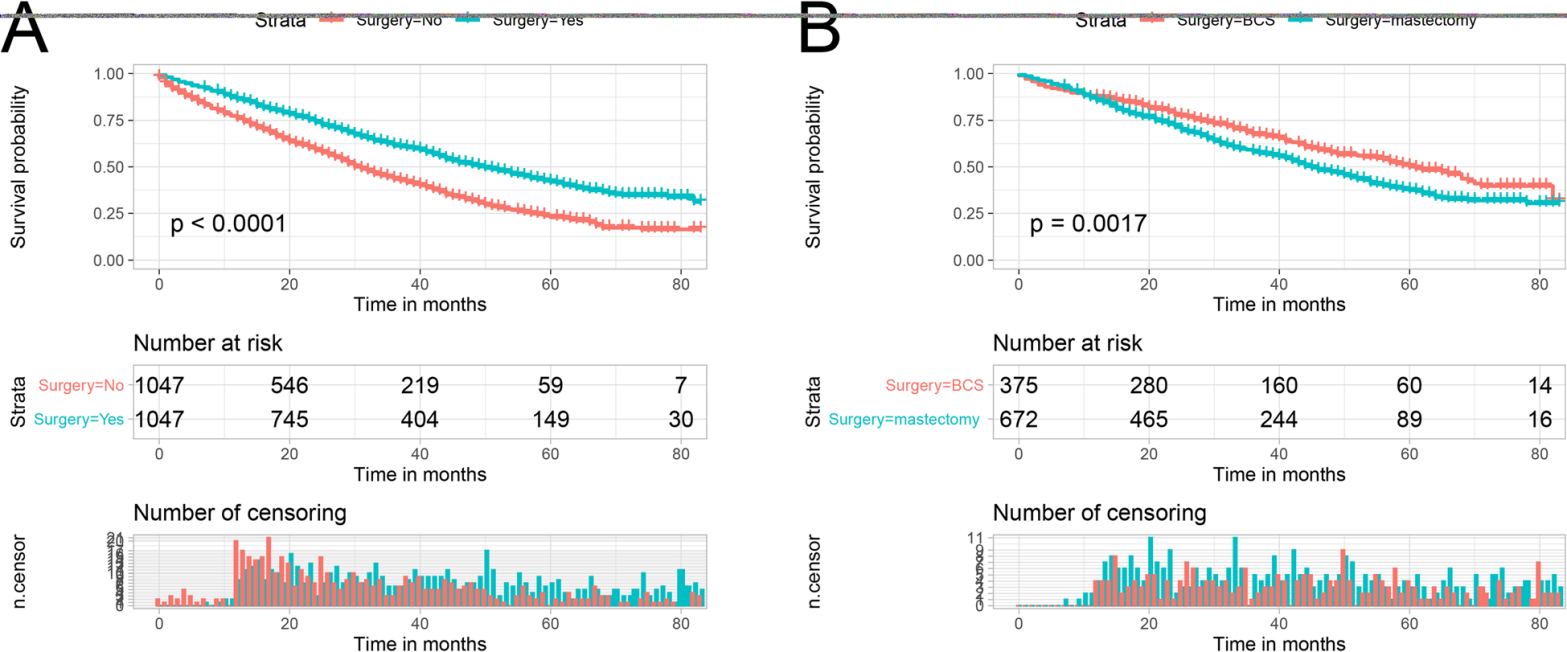
Kaplan-Meier curves for OS. **a** Overall analysis of OS: Surgery group vs. non-surgery, **b** Overall analysis of OS: BCS subgroup vs. mastectomy subgroup. OS: Overall survival

The Kaplan-Meier curves for OS in the surgery and non- surgery groups after PSM are shown in Fig. 2a. Surgery at the primary site significantly improved OS in breast cancer patients with BM, with a median survival of 50 months in the surgery group versus 31 months in the non-surgery group (P<0.001). Furthermore, we further analyzed the impact of the type of surgery on the OS of breast cancer patients with BM. As shown in Fig. 2b, for the OS of patients, BCS improved more significantly compared to mastectomy (median OS: 61 months vs. 45 months, P<0.05).

### Development and validation of a prognostic nomogram for patients in the surgery group before PSM

A total of 1454 patients in the surgery group were randomized in a 7:3 ratio into the training cohort (1020) and validation cohort (434). To identify independent prognostic factors in the surgery group, univariate Cox analysis was performed on the training cohort. Age, race, histological type, grade, T stage, N stage, type of surgery, radiotherapy, chemotherapy, brain metastasis, liver metastasis, lung metastasis, tumor size, breast subtype, and marital status were found to be important factors affecting the OS (Table 2). After controlling for confounding variables with multivariate Cox analysis, age, race, histological type, grade, N stage, type of surgery, chemotherapy, brain metastasis, liver metastasis, lung metastasis, tumor size, and breast subtype were identified as independent prognostic factors (Table 2).

**Table 2:** Univariate and multivariate Cox analysis for BC patients with BM in surgery group

	Univariate Cox analysis				Multivariate Cox analysis			
	HR	95%CI		P	HR	95%CI		P
Age								
< 51	1				1			
51–78	1.344	1.087	1.662	0.006	1.320	1.054	1.653	0.016
> 78	3.250	2.367	4.463	0.000	3.038	2.129	4.336	0.000
Race								
Black	1				1			
Other	0.643	0.421	0.982	0.041	0.642	0.417	0.990	0.045
White	0.682	0.537	0.866	0.002	0.712	0.554	0.915	0.008
Sex								
Female	1							
Male	1.486	0.838	2.637	0.176				
Primary site								
Central portion	1							
Upper-inner	0.998	0.661	1.506	0.993				
Lower-inner	1.271	0.794	2.034	0.317				
Upper-outer	1.121	0.810	1.552	0.492				
Lower-outer	0.877	0.562	1.366	0.561				
Others	1.025	0.737	1.425	0.885				
Laterality								
Left	1							
Right	0.908	0.759	1.086	0.290				
Histological type								
Ductal	1				1			
Lobular	1.048	0.838	1.311	0.681	1.083	0.845	1.387	0.528
Others	1.774	1.164	2.705	0.008	1.891	1.231	2.905	0.004
Grade								
I	1				1			
II	1.187	0.800	1.762	0.395	1.307	0.874	1.955	0.192
III	1.959	1.337	2.871	0.001	1.968	1.313	2.952	0.001
IV	8.574	2.036	36.105	0.003	4.009	0.909	17.675	0.067
T stage								
T1	1							
T2	1.750	1.217	2.516	0.003				
T3	2.355	1.612	3.439	0.000				
T4	2.163	1.477	3.168	0.000				
N stage								
N0 + N1	1				1			
N2 + N3	1.358	1.136	1.623	0.001	1.221	1.010	1.477	0.039
Surgery								
BCS	1				1			
Mastectomy	1.466	1.201	1.789	0.000	1.253	1.007	1.558	0.043
Radiotherapy								
No	1							
Yes	0.813	0.680	0.972	0.023				
Chemotherapy								
No	1				1			
Yes	0.774	0.646	0.929	0.006	0.729	0.585	0.909	0.005
Brain metastasis								
No	1				1			
Yes	2.177	1.359	3.488	0.001	2.694	1.656	4.382	0.000
Liver metastasis								
No	1				1			
Yes	1.993	1.585	2.506	0.000	2.207	1.725	2.824	0.000
Lung metastasis								
No					1			
Yes	1.647	1.314	2.066	0.000	1.274	1.003	1.619	0.047
Tumor size, cm								
< 2	1				1			
2–5	1.665	1.145	2.420	0.008	1.370	0.938	2.003	0.104
> 5	2.504	1.716	3.653	0.000	1.818	1.223	2.703	0.003
Breast subtype								
HR−/HER2+	1				1			
HR+/HER2-	1.414	0.899	2.223	0.134	1.770	1.097	2.854	0.019
HR+/HER2+	1.052	0.634	1.746	0.845	1.160	0.691	1.946	0.574
HR−/HER2-	3.584	2.203	5.832	0.000	3.603	2.185	5.943	0.000
Insurance status								
Uninsured	1							
Insured	0.621	0.365	1.057	0.079				
Marital status								
Unmarried	1							
Married	0.743	0.622	0.888	0.001				

**Fig. 3:**
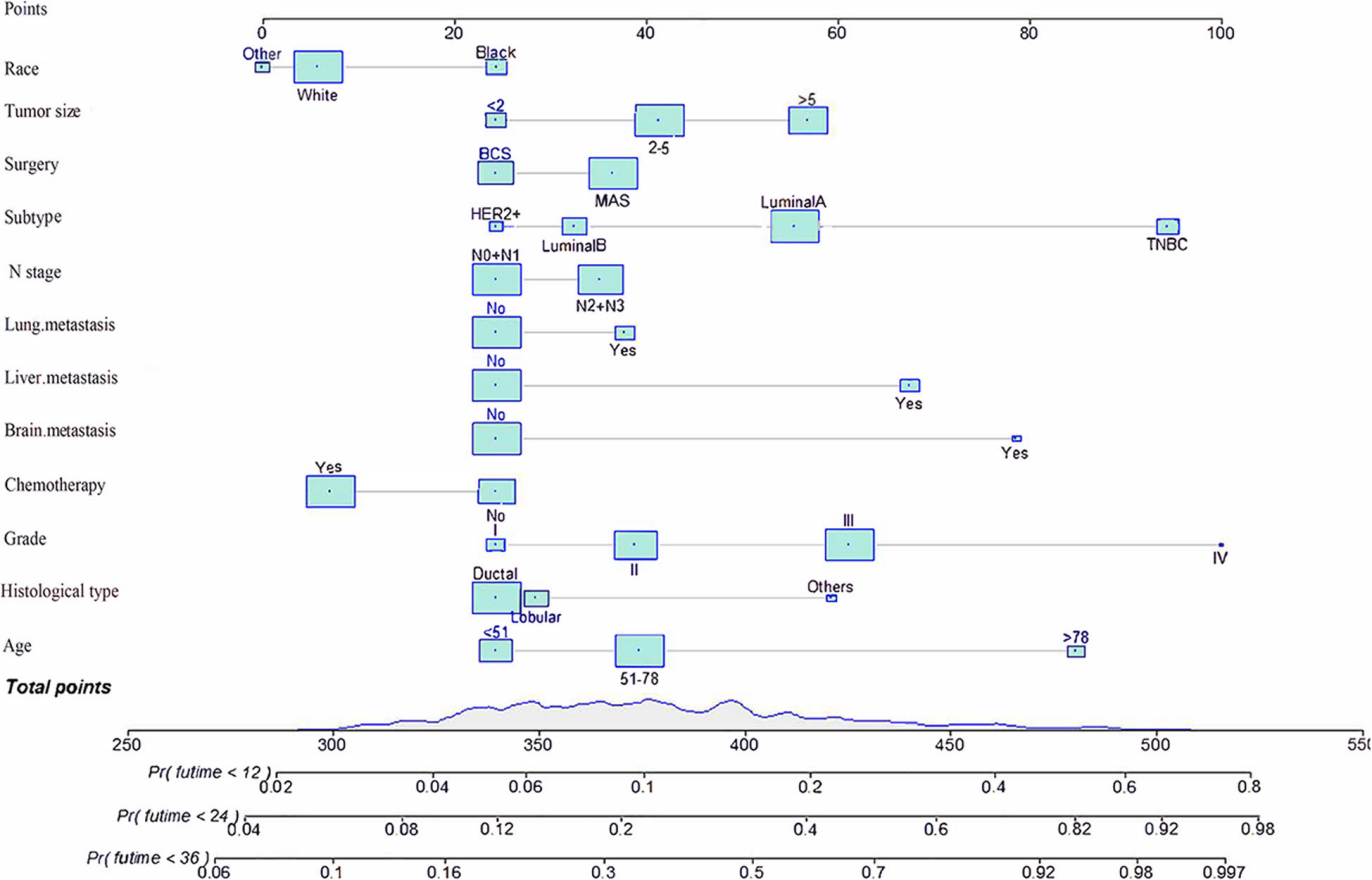
Prognostic nomogram for patients with surgery on primary tumor

**Fig. 4:**
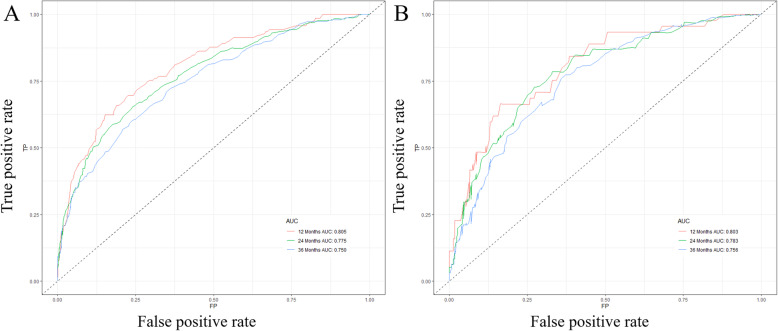
ROC curves for survival prediction of patients with surgery on primary tumor. **a** ROC curves of 12-, 24-, and 36-months in the training cohort, **b** ROC curves of 12-, 24-, and 36-months in the validation cohort. ROC: Receiver operating characteristic

**Fig. 5:**
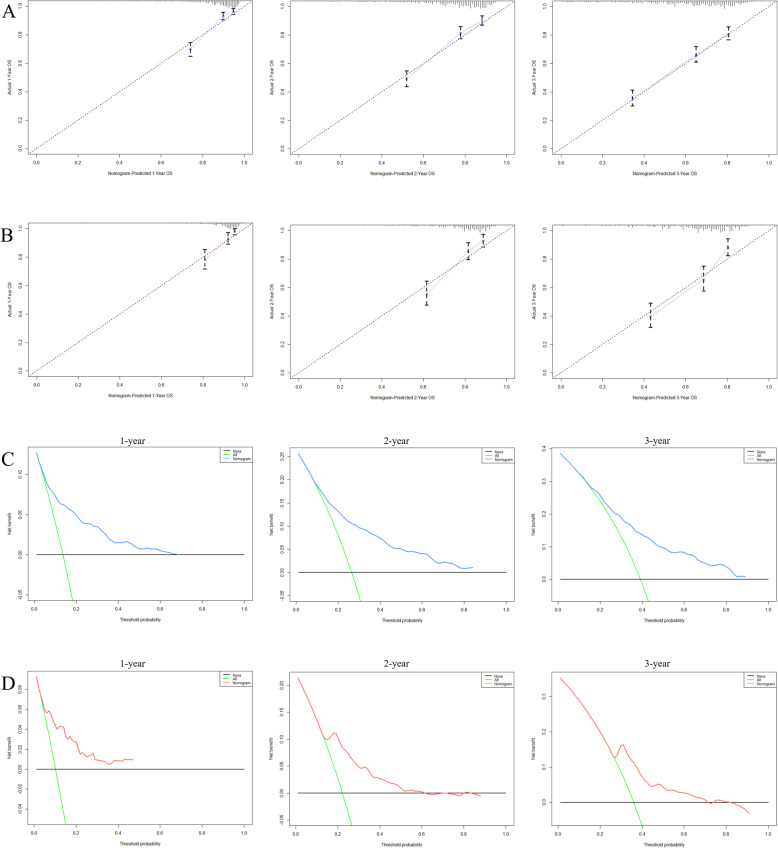
Calibration curves and DCA for survival prediction of patients with surgery on primary tumor. **a** Calibration curves of 12-, 24-, and 36-months in the training cohort, **b** Calibration curves of 12-, 24-, and 36-months in the validation cohort, **c** DCA of 12-, 24-, and 36-months in the training cohort, **d** DCA of 12-, 24-, and 36-months in the validation cohort. DCA: Decision curve analysis

**Fig. 6:**
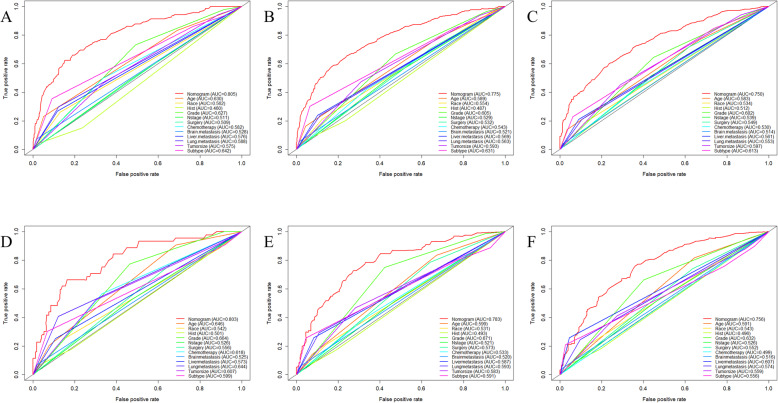
ROC curves for surgery group. The ROC curves of nomogram and all independent predictors at 12- (**a**), 24- (**b**), and 36-months (**c**) in the training cohort and at 12- (**d**), 24- (**e**), and 36-months (**f**) in the validation cohort. ROC: Receiver operating characteristic

*BC* breast cancer, *BM* bone metastases,

Based on the prognostic factors selected in the training cohort, a nomogram was constructed for predicting 1-, 2-, and 3-year OS of patients underwent surgery (Fig. 3). Subsequently, the discrimination of the nomogram was verified by plotting the ROC curves. The AUC values for predicting 1-, 2-, and 3-year OS were 0.805, 0.775, and 0.750 in the training cohort and 0.803, 0.783, and 0.756 in the validation cohort (Fig. 4). Furthermore, the calibration curve and DCA that in both the training cohort and the validation cohort indicated that the nomogram not only showed a high agreement between the predicted OS and the actual outcome (Fig. 5a and b) but also showed a significant positive net benefit across a wide range of mortality risks, demonstrating that the nomogram has a strong clinical utility (Fig. 5c and d). Besides, we further compared the differences of AUC values between the nomograms and all independent prognostic factors. The results showed that the AUC values of nomograms were higher than the AUC values of all independent factors at 1-, 2-, and 3-years in both the training and validation cohorts (Fig. 6).

**Fig. 7:**
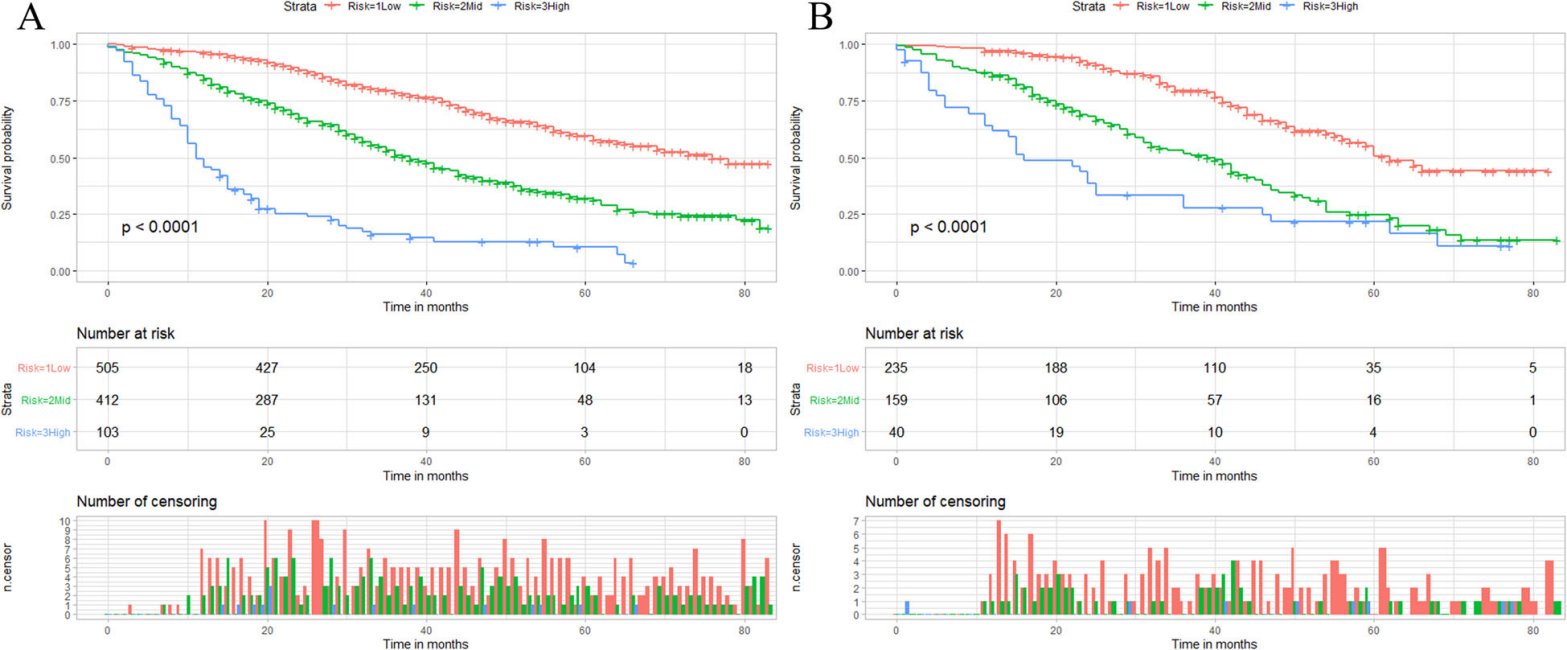
X-tile analysis and Kaplan-Meier curves for the training cohort (**a**) and validation cohort (**b**) OS of the surgery group. OS: Overall survival

We calculated the total score of the training cohort of patients based on the nomogram. The best OS-based cutoffs for the total score were determined by X-tile software and were 373 and 435, respectively. Therefore, we specify that less than 373 is classified as a low mortality risk subgroup, greater than 435 as a high mortality risk subgroup, and 373 to 435 as a middle mortality risk subgroup. Kaplan-Meier curves showed that in both training and validation cohorts, patients in the low mortality risk subgroup have a better prognosis than those in the middle mortality risk subgroup, and patients in the middle mortality risk subgroup have a better prognosis than those in the high mortality risk subgroup (P<0.01, Fig. 7). Patients who are classified as a low risk of death subgroup can derive the greatest survival benefit from the surgery.

### Development and validation of a prognostic nomogram for patients in the non-surgery group before PSM

Randomization of the non-surgery group at a 7:3 ratio resulted in 1753 patients being enrolled in the training cohort and 749 patients being enrolled in the validation cohort. All results from univariate and multivariate Cox analyses in the training cohort are shown in Table 3. The univariate Cox analysis showed that age, race, histological type, grade, chemotherapy, brain metastasis, liver metastasis, lung metastasis, breast subtype, and marital status were significantly associated with OS (*p*-value < 0.05). Subsequently, we performed multivariate Cox analysis on variables that were meaningful in univariate Cox analysis. Unexpectedly, the 10 variables previously shown in univariate Cox analyses to be significantly associated with OS were identified as independent prognostic factors.

**Table 3:** Univariate and multivariate Cox analysis for BC patients with BM in non-surgery group

	Univariate Cox analysis				Multivariate Cox analysis			
	HR	95%CI		P	HR	95%CI		P
Age								
< 51	1				1			
51–78	1.421	1.219	1.657	0.000	1.425	1.218	1.667	0.000
> 78	2.247	1.827	2.764	0.000	2.300	1.839	2.875	0.000
Race								
Black	1				1			
Other	0.732	0.558	0.960	0.024	0.803	0.609	1.059	0.120
White	0.807	0.687	0.948	0.009	0.795	0.673	0.939	0.007
Sex								
Female	1							
Male	1.139	0.659	1.968	0.641				
Primary site								
Central portion	1							
Upper-inner	1.118	0.840	1.487	0.444				
Lower-inner	1.237	0.910	1.681	0.174				
Upper-outer	1.006	0.804	1.259	0.957				
Lower-outer	1.004	0.738	1.367	0.978				
Others	1.122	0.894	1.408	0.322				
Laterality								
Left	1							
Right	1.034	0.916	1.168	0.584				
Histological type								
Ductal	1				1			
Lobular	1.043	0.881	1.236	0.622	1.239	1.038	1.479	0.018
Others	1.651	1.292	2.109	0.000	1.450	1.131	1.859	0.003
Grade								
I	1				1			
II	1.177	0.946	1.464	0.144	1.289	1.032	1.610	0.025
III	1.662	1.332	2.073	0.000	1.634	1.291	2.067	0.000
IV	2.917	1.354	6.287	0.006	2.295	1.049	5.021	0.037
T stage								
T1	1							
T2	0.880	0.732	1.058	0.174				
T3	0.928	0.753	1.145	0.487				
T4	1.043	0.861	1.262	0.667				
N stage								
N0 + N1	1							
N2 + N3	1.097	0.936	1.286	0.254				
Radiotherapy								
No	1							
Yes	1.116	0.986	1.264	0.084				
Chemotherapy								
No	1				1			
Yes	0.855	0.757	0.966	0.012	0.710	0.613	0.823	0.000
Brain metastasis								
No	1				1			
Yes	2.113	1.715	2.604	0.000	1.763	1.418	2.192	0.000
Liver metastasis								
No					1			
Yes	1.569	1.373	1.793	0.000	1.601	1.378	1.859	0.000
Lung metastasis								
No					1			
Yes	1.403	1.231	1.601	0.000	1.232	1.074	1.413	0.003
Tumor size, cm								
< 2	1							
2–5	0.934	0.781	1.116	0.450				
> 5	1.016	0.841	1.228	0.867				
Breast subtype								
HR−/HER2+	1				1			
HR+/HER2-	0.827	0.635	1.076	0.157	0.872	0.656	1.160	0.348
HR+/HER2+	0.726	0.538	0.981	0.037	0.713	0.527	0.966	0.029
HR−/HER2-	3.412	2.500	4.657	0.000	3.320	2.419	4.557	0.000
Insurance status								
Uninsured	1							
Insured	1.002	0.758	1.324	0.990				
Marital status								
Unmarried	1				1			
Married	0.770	0.681	0.872	0.000	0.850	0.748	0.965	0.012

*BC* breast cancer, *BM* bone metastases

**Fig. 8:**
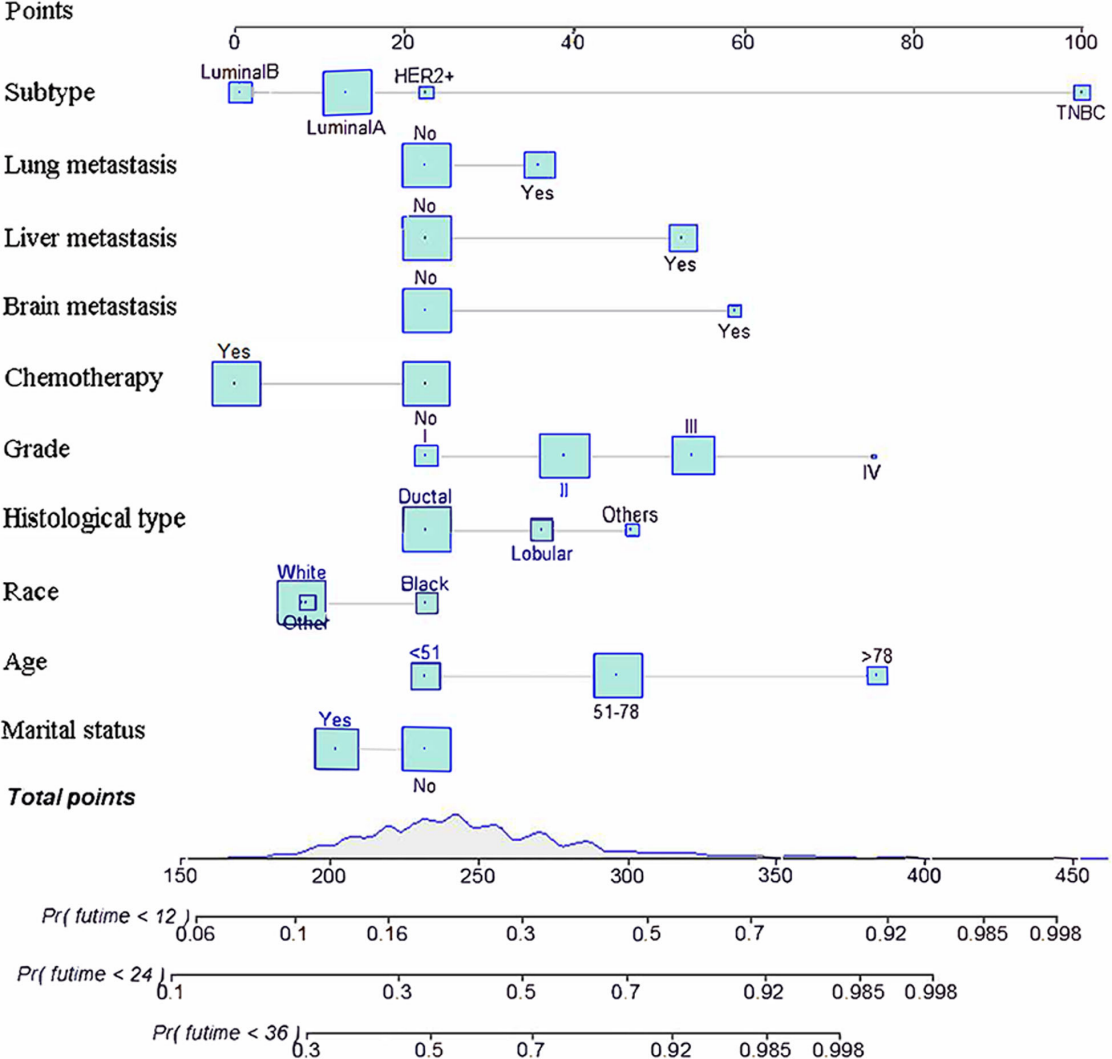
Prognostic nomogram for patients without surgery on primary tumor

**Fig. 9:**
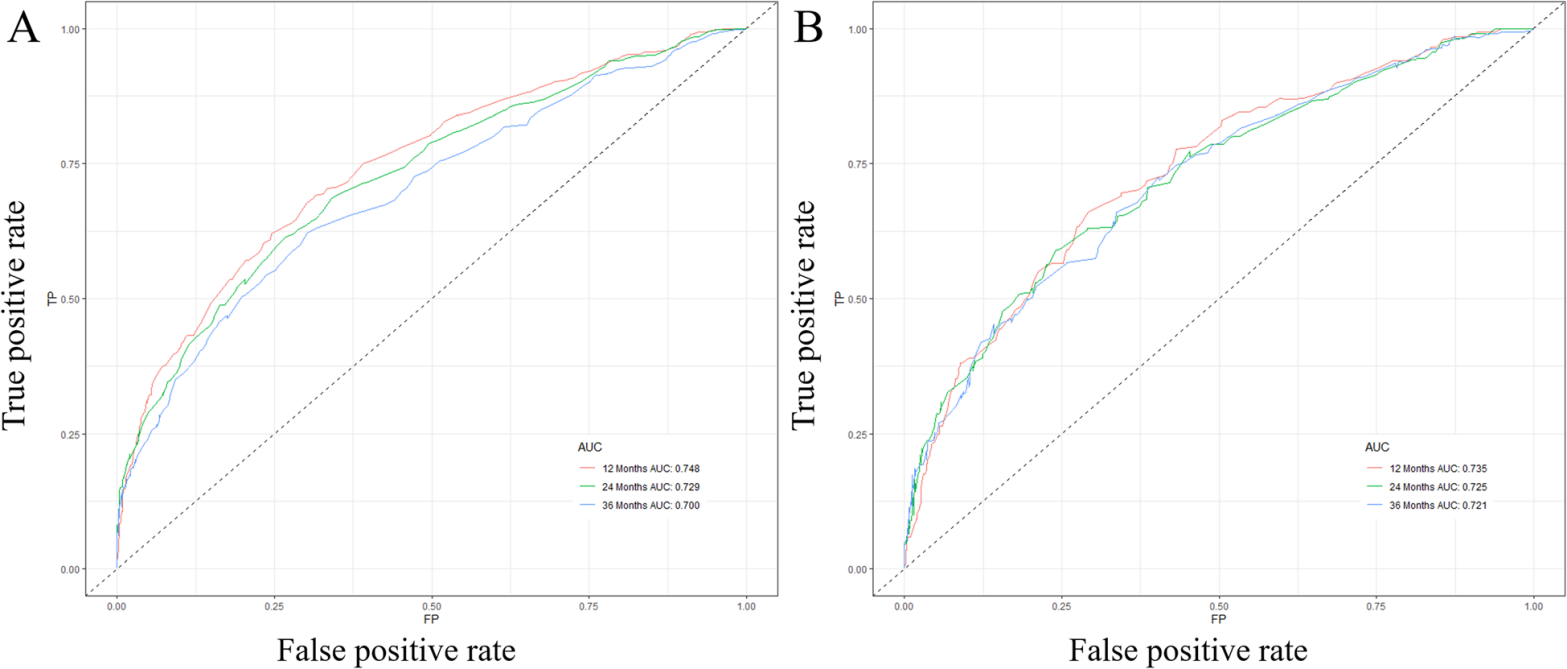
ROC curves for survival prediction of patients without surgery on primary tumor. **a** ROC curves of 12-, 24-, and 36-months in the training cohort, **b** ROC curves of 12-, 24-, and 36-months in the validation cohort. ROC: Receiver operating characteristic

**Fig. 10:**
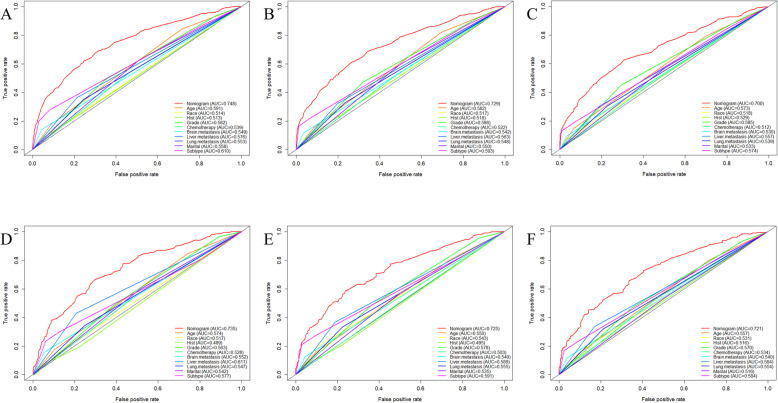
ROC curves for non-surgery group. The ROC curves of nomogram and all independent predictors at 12- (**a**), 24- (**b**), and 36-months (**c**) in the training cohort and at 12- (**d**), 24- (**e**), and 36-months (**f**) in the validation cohort. ROC: Receiver operating characteristic

**Fig. 11:**
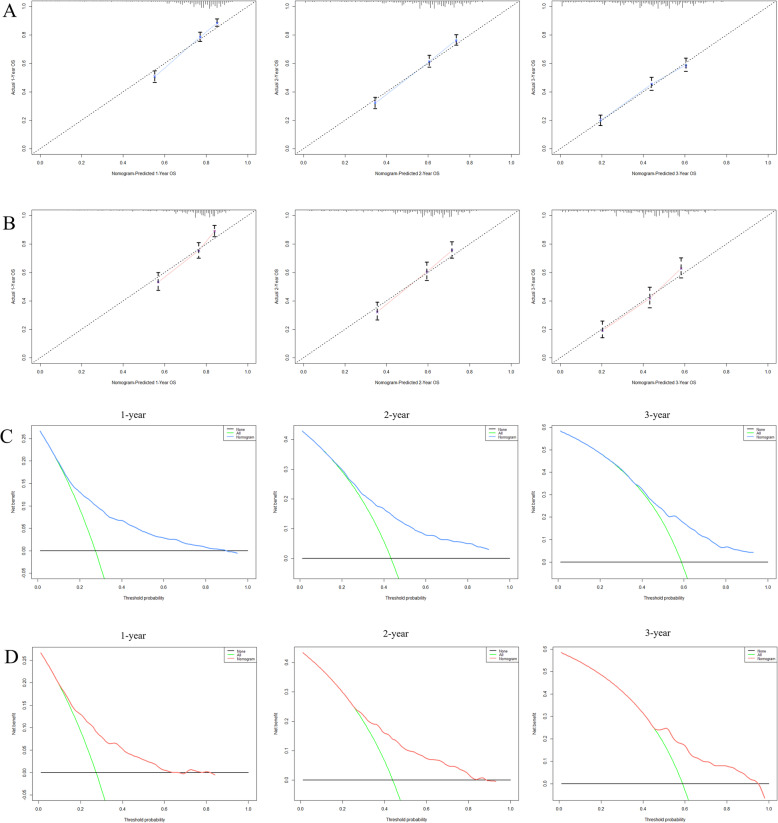
Calibration curves and DCA for survival prediction of patients without surgery on primary tumor. **a** Calibration curves of 12-, 24-, and 36-months in the training cohort, **b** Calibration curves of 12-, 24-, and 36-months in the validation cohort, **c** DCA of 12-, 24-, and 36-months in the training cohort, **d** DCA of 12-, 24-, and 36-months in the validation cohort. DCA: Decision curve analysis

A nomogram was constructed to predict the OS at 1-, 2-, and 3- years in the non-surgery group based on independent prognostic factors (Fig. 8). The time-dependent ROCs showed that the nomogram not only performs excellently in predicting OS (Fig. 9) but also has a higher prediction accuracy than a single independent prognostic factor (Fig. 10). Observation of the calibration curves of the nomogram showed, unsurprisingly, that there was a high degree of agreement between the predicted and actual results in the training and validation cohorts (Fig. 11a and b). Moreover, the DCA also demonstrated the strong clinical applicability of the nomogram model for the non-surgery group (Fig. 11c and d).

**Fig. 12:**
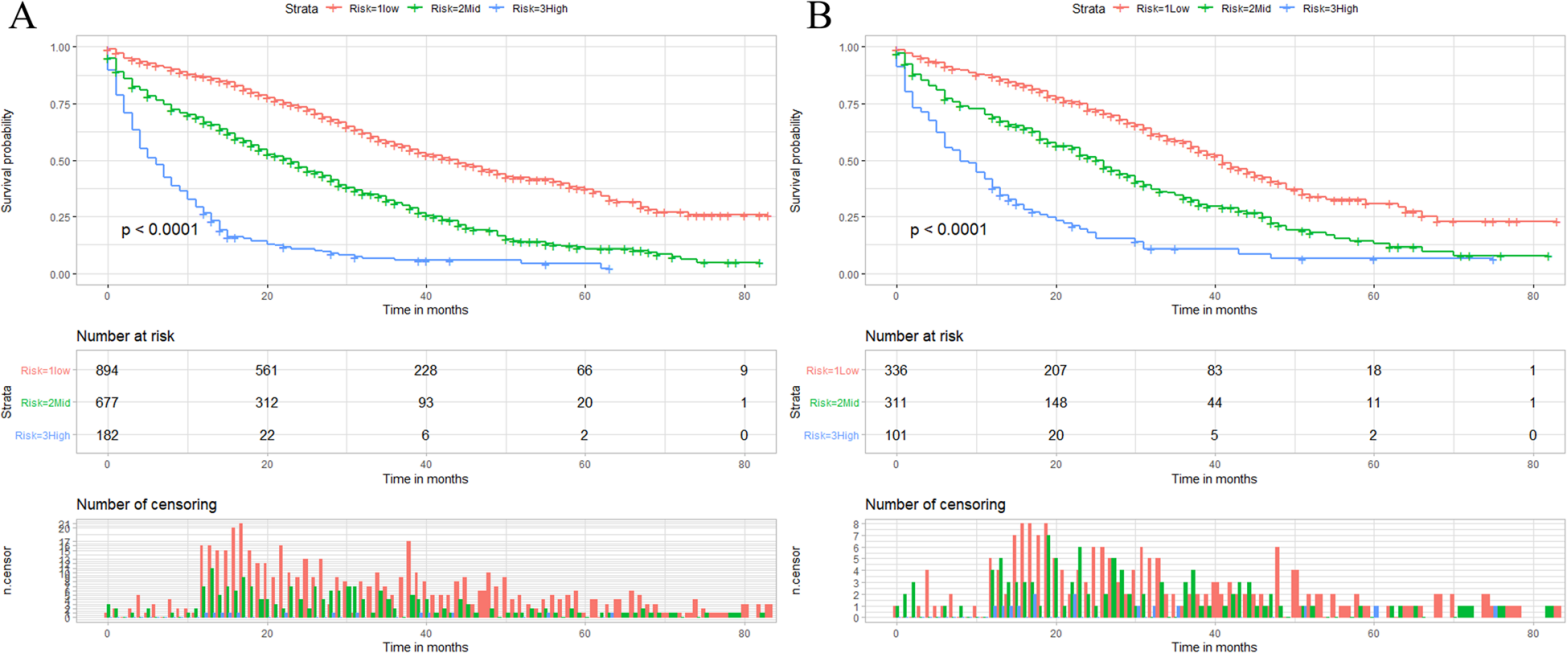
X-tile analysis and Kaplan-Meier curves for the training cohort (**a**) and validation cohort (**b**) OS of the non-surgery group. OS: Overall survival

We categorized the non-surgery group of patients into low mortality risk subgroups, middle mortality risk subgroups, and high mortality risk subgroups by X-tile software. Patients with scores below 247 were classified in the low mortality risk subgroup, those above 302 were classified in the high mortality risk subgroup, and those between 247 and 302 were classified in the middle mortality risk subgroup. Interestingly, as shown in Fig. 12, we found that as with the subgroup analysis of patients in the surgery group, when patients were classified in the low mortality risk subgroup, it always meant a better prognosis.

## Discussion

As metastatic breast cancer is still considered incurable, the primary goal of treatment is to extend life expectancy and improve the quality of life. According to NCCN guidelines, the current primary treatment for patients with metastatic breast cancer is systemic therapy, rather than recommending surgery [7]. Several retrospective studies have shown that surgery of the primary tumor can provide a survival benefit for patients with metastatic breast cancer [19–21].. Nevertheless, it is only by focusing on a more specific and limited disease that the potential benefits of surgery can be better understood and individualized treatment strategies developed. In the present study, we determined that surgery of the primary tumor has a positive effect in improving the prognosis of breast cancer patients with BM. Moreover, we developed prognostic nomograms to predict OS at 1-, 2-, and 3-years in patients who underwent surgery and those who did not. The high predictive accuracy and clinical utility of nomograms were demonstrated by developing ROC, calibration curves, and DCA. For both clinicians and patients, this can be a useful clinical decision-making tool.

To date, the role of primary tumor surgery in the treatment of breast cancer patients with BM remains unclear and there is no consensus. The prognostic role of surgery in patients with stage IV or distant metastases of breast cancer has been reported in many previous studies, however, there is no uniform conclusion [22–25]. A retrospective study by Jennifer et al. compared the OS rates of women who underwent surgery with those who did not, and the multivariate analysis showed that patients with stage IV breast cancer who underwent surgery had a significantly longer median survival than those who did not (*P* < 0.001, [26]. In a separate phase III randomized controlled trial, the impact of surgery for the primary tumor on survival was evaluated in patients with stage IV breast cancer. After 3 years follow-up, no survival advantage was obtained for surgery. However, after 5 years of follow-up, OS was better in the surgery group (HR = 0.66, 95% CI [0.49–0.88]; *p* = 0.005), and subgroup analysis showed that the survival benefit of surgery was demonstrated in patients who were younger (< 55 years), ER/PR positive, HER2 negative, or had only BM [27]. Surgical resection of the primary tumor can reduce the number of circulating tumor cells, thereby reducing the tumor burden and potentially reversing tumor-induced immunosuppression and preventing the development of an impaired immune state [28, 29]. However, a limited number of prospective randomized controlled clinical trials have produced conflicting results. A recently published study in Austria indicates no OS benefit of surgical resection of the primary tumor in primary stage IV breast cancer [30]. No credible conclusions can be drawn from these studies because they are small and non-randomized, and all of them have cohorts selected from a single institution. In our study, we focused on a more specific type of metastatic breast cancer, as breast cancer patients with BM have longer survival in metastatic breast cancer and it has been reported that patients with BM have more circulating tumor cells in their blood than patients without BM [31, 32]. Thus, breast cancer patients with BM were considered as our preferred study subjects. Meanwhile, we utilized PSM to reduce the bias between the surgery and non-surgery groups, making our conclusions more accurate and convincing. For breast cancer patients with BM, surgery of the primary tumor can provide survival benefits, which is a departure from the previous perception that surgery of the primary tumor is only for patients with early-stage breast cancer. What is surprising is that by Kaplan-Meier analysis and the log-rank test, we also found that BCS can provide greater survival benefits to breast cancer patients with BM compared to mastectomy (Fig. 2b).

Whether to undergo mastectomy or BCS is often one of the most difficult decisions for breast cancer patients, and we have found that the surgical approach is an equally important factor in the prognosis of breast cancer patients with BM. The long-term survival of women undergoing BCS is similar to that of women undergoing mastectomy, according to a recent randomized controlled trial [33]. Considering the post-operative impact on the quality of life and advances in medical technology, BCS is more acceptable to patients. A study based on the SEER database concluded that BCS plus radiotherapy has a better prognosis than mastectomy [34]. However, these studies were conducted on patients with early-stage breast cancer. BCS, we have found, is equally significant for breast cancer patients with BM. The absence of breasts after a mastectomy has a significant impact on a patient’s quality of life, always reminding them of the fact that they are breast cancer patients. For most people, BCS produces acceptable cosmetic results and is not only safe compared to mastectomy, but also reduces psychological morbidity, significantly reduces anxiety and depression, and improves body image, sexual behavior, and self-esteem [35]. Depending on the results of this study, BCS can be recommended for breast cancer patients with BM, which not only provides greater survival benefits but also is more acceptable to patients.

To the best of our knowledge, this is the first study to construct nomograms predicting the prognosis of breast cancer patients with BM based on large and diverse case data. Nomograms are considered an effective tool for quantifying risk and maximizing forecast accuracy [36, 37]. The calibration curves showed a high degree of agreement between the predicted and actual observed survival rates of the training and validation cohorts, indicating that the nomograms established in this study are reliable. We developed univariate and multivariate Cox regression models in the surgery and non-surgery groups, respectively, to identify risk factors associated with survival. The results indicated that age, race, histological type, grade, chemotherapy, distant metastatic site, and breast subtype were independent risk factors for OS in the surgery and non-surgery groups, which is consistent with previous studies [38]. Marital status, interestingly, was an independent prognostic factor in the non-surgery group and suggested that unmarried patients were at higher risk of poor prognosis. It is consistent with the findings of a recent systematic review that showed that unmarried patients are at higher risk of metastatic cancer and have shorter survival times [39]. These trends are likely due to the lack of positive influence of marriage, reducing the likelihood that cancer will be diagnosed at an earlier stage while demonstrating the potentially significant impact of social support on cancer detection, treatment, and survival.

Inevitably, some limitations of our study exist. First, some patients were excluded due to missing data, which may have led to selection bias. Second, the SEER database provides information on the surgical site, but not detailed surgery-related information on indications, intraoperative bleeding, complications, and so on. In addition, the SEER database does not have detailed information on the number of metastases, tumor resection residues, targeted therapy regimens, chemotherapy regimens, and endocrine therapy, which can lead to study bias.

## Conclusion

In summary, this study demonstrates the potential survival benefit of surgery for primary tumors in breast cancer patients with BM by analyzing population-based data. Furthermore, these nomograms constructed in this study may be important and effective models for predicting the OS of breast cancer patients with BM. For both clinicians and patients, the risks and benefits of surgery can be better weighed, providing new ideas for the comprehensive treatment of breast cancer patients with BM.

## Data Availability

The data of this study are from SEER database.

## Abbreviations

BM: Bone metastases
SEER: Surveillance, Epidemiology, and End Results
OS: Overall survival
BCS: Breast-conserving surgery
PSM: Propensity score matching
ROC: Receiver operating characteristic
AUC: Area under the curve
DCA: Decision curve analysis.
